# 2-Iodo-3-(4-meth­oxy­anilino)-5,5-dimethyl­cyclo­hex-2-en-1-one

**DOI:** 10.1107/S1600536812002255

**Published:** 2012-01-25

**Authors:** S. Paramasivam, G. Bhaskar, P. R. Seshadri, P. T. Perumal

**Affiliations:** a Post Graduate and Research Department of Physics, Agurchand Manmull Jain College, Chennai 600 114, India; b Organic Chemistry Division, Central Leather Research Institute, Chennai 600 020, India

## Abstract

The cyclo­hexene ring in the title compound, C_15_H_18_INO_2_, adopts a sofa conformation. The dihedral angle between the cyclo­hexene (through all ring atoms) and benzene rings is 63.3 (1)°. The mol­ecular conformation features an N—H⋯I short contact and the crystal packing features C—H⋯O hydrogen bonds.

## Related literature

For the biological activity of cyclo­hex-2-enone derivatives, see: Correia *et al.* (2001 ▶); Rebacz *et al.* (2007 ▶); Stadler *et al.* (1994 ▶). For the use of cyclo­hex-2-enone in organic synthesis, see: Cokcer *et al.* (1995 ▶); Pandey *et al.* (2004 ▶). For pukering parameters, see: Cremer & Pople, (1975 ▶). For related structures, see: Mohan *et al.* (2008 ▶); North *et al.* (2011 ▶).
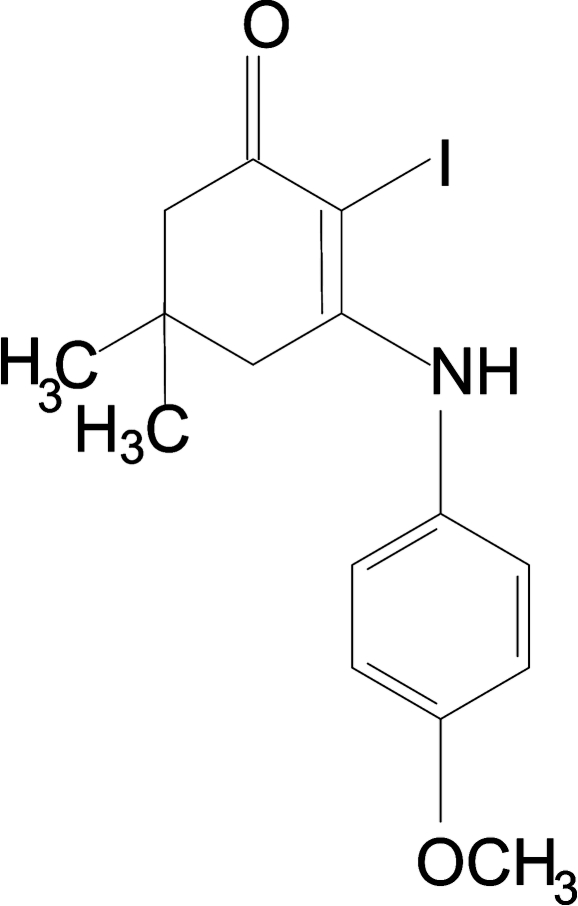


## Experimental

### 

#### Crystal data

C_15_H_18_INO_2_
*M* *_r_* = 371.20
Orthorhombic, 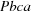

*a* = 15.922 (5) Å
*b* = 10.107 (5) Å
*c* = 19.034 (5) Å
*V* = 3063 (2) Å^3^
*Z* = 8
Mo *K*α radiation
μ = 2.09 mm^−1^
*T* = 298 K
0.20 × 0.20 × 0.20 mm

#### Data collection

Bruker SMART APEXII area-detector diffractometer
15382 measured reflections
3785 independent reflections
2793 reflections with *I* > 2σ(*I*)
*R*_int_ = 0.025

#### Refinement

*R*[*F* ^2^ > 2σ(*F* ^2^)] = 0.027
*wR*(*F* ^2^) = 0.074
*S* = 0.93
3785 reflections
172 parameters
H-atom parameters constrained
Δρ_max_ = 0.46 e Å^−3^
Δρ_min_ = −0.53 e Å^−3^

### 

Data collection: *APEX2* (Bruker, 2008 ▶); cell refinement: *SAINT* (Bruker, 2008 ▶); data reduction: *SAINT*; program(s) used to solve structure: *SHELXS97* (Sheldrick, 2008 ▶); program(s) used to refine structure: *SHELXL97* (Sheldrick, 2008 ▶); molecular graphics: *ORTEP-3* (Farrugia, 1997 ▶) and *PLATON* (Spek, 2009 ▶); software used to prepare material for publication: *SHELXL97*, *PLATON* and *publCIF* (Westrip, 2010 ▶).

## Supplementary Material

Crystal structure: contains datablock(s) I, global. DOI: 10.1107/S1600536812002255/kp2383sup1.cif

Structure factors: contains datablock(s) I. DOI: 10.1107/S1600536812002255/kp2383Isup2.hkl

Supplementary material file. DOI: 10.1107/S1600536812002255/kp2383Isup3.cml

Additional supplementary materials:  crystallographic information; 3D view; checkCIF report

## Figures and Tables

**Table 1:** Hydrogen-bond geometry (Å, °)

*D*—H⋯*A*	*D*—H	H⋯*A*	*D*⋯*A*	*D*—H⋯*A*
C14—H14⋯O1^i^	0.93	2.39	3.313 (3)	174
N1—H1⋯I1	0.86	2.71	3.227 (2)	120

Symmetry code: (i) 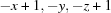.

**Fig. 1.**
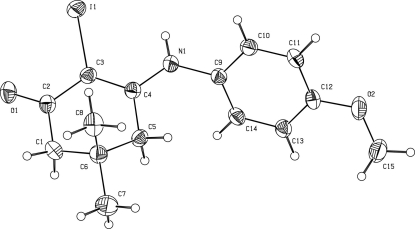
The molecular structure of the title compound showing the atom-numbering scheme and displacement ellipsoids drawn at the 30% probability level.

**Fig. 2.**
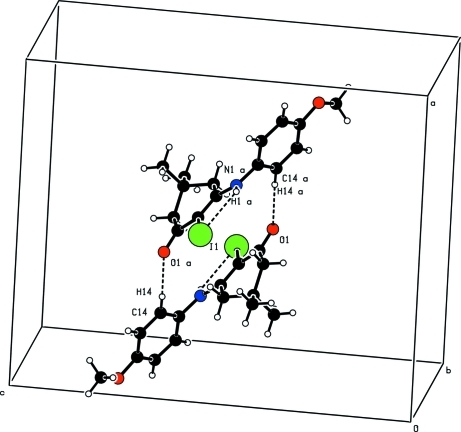
The crystal packing of the title compound. Hydrogen bonds are shown by dashed lines.

## supplementary crystallographic information

### Comment

Cyclohex-2-enone derivative exhibits antibacterial (Stadler *et al.*, 1994) and anticancer (Correia *et al.*, 2001; Rebacz *et al.*, 2007) activities. Cyclohex-2-enone plays an important role in organic synthesis (Cokcer *et al.*, 1995; Pandey *et al.*, 2004). Against this background, the title compound was chosen for X-ray structure analysis (Fig. 1). The cyclohexene ring adopts a sofa conformation with the pukering parameters (Cremer & Pople, 1975) being q_2_=0.409 (3) Å, q_3_=-0.247 (3)Å and Q_T_=0.478 (3) Å. The molecular structure is stabilised by N—H···I intramolecular interactions and the crystal packing is stabilised by C—H···O hydrogen bonds (Fig. 2 and Table 1).

### Experimental

1,3-cyclohexanedione (2 mmol), FeCl_3_.6H_2_O (5 mol), and 50 mg of sodium sulfate were succesively added in a dry Schlenk tube under argon. The solids were then dissolved in 3 mL of dichloromethane and stirred for 5 m. Aniline (2 mmol) was slowly added and the dark brown cloloured mixture was allowed to stir overnight. After completion, solvents were removed under vacuum and the crude oil was filtered on a plug of neutral alumina (eluent: dichloromethane/ methanol, 90/10). Solvents were then removed and enaminone product was obtained as a bright yellow solid. Then iodine (3 mmol) dissolved in CCl_4_/pyridine (1;1, 10 mL) was added dropwise under an atmosphere of argon to a solution of enaminone (1.5 mmol) in CCl_4_/pyridine (1;1, 10 mL) at 273 K. The mixture was stirred for 2 h during that time the temperature was allowed to raise to room temperature. The mixture was diluted with ethyl acetate (50 mL) and washed successively with 1 NHCl (4× 10 mL), sat. NaHCo_3_ (20 mL), 20% aqueous Na_2_S_2_O_3_ (20 mL) and dried (Na_2_SO_4_). Filtered and concentrated under reduced pressure, the residue was further purified by column chromatography to afford pure 2-iodo-5,5-dimethyl-3-(Phenylamino) cyclohex-2-enone.

### Refinement

Hydrogen atoms were positioned geometrically and allowed to ride on their parent atoms, with C—H = 0.93 - 0.97 Å and *U*_iso_(H) = 1.5*U*_eq_(C) for methyl H atoms and 1.2 *U*_eq_(C) for other H atoms.

### Crystal data

**Table:**

C_15_H_18_INO_2_	*F*(000) = 1472
*M**_r_* = 371.20	*D*_x_ = 1.610 Mg m^−^^3^
Orthorhombic, *P**b**c**a*	Mo *K*α radiation, λ = 0.71073 Å
Hall symbol: -P 2ac 2ab	Cell parameters from 3785 reflections
*a* = 15.922 (5) Å	θ = 2.1–28.3°
*b* = 10.107 (5) Å	µ = 2.09 mm^−^^1^
*c* = 19.034 (5) Å	*T* = 298 K
*V* = 3063 (2) Å^3^	Block, colourless
*Z* = 8	0.20 × 0.20 × 0.20 mm

### Data collection

**Table:**

Bruker SMART APEXII area-detector diffractometer	2793 reflections with *I* > 2σ(*I*)
Radiation source: fine-focus sealed tube	*R*_int_ = 0.025
graphite	θ_max_ = 28.3°, θ_min_ = 2.1°
ω and φ scans	*h* = −20→21
15382 measured reflections	*k* = −13→13
3785 independent reflections	*l* = −18→25

### Refinement

**Table:**

Refinement on *F*^2^	Primary atom site location: structure-invariant direct methods
Least-squares matrix: full	Secondary atom site location: difference Fourier map
*R*[*F*^2^ > 2σ(*F*^2^)] = 0.027	Hydrogen site location: inferred from neighbouring sites
*wR*(*F*^2^) = 0.074	H-atom parameters constrained
*S* = 0.93	*w* = 1/[σ^2^(*F*_o_^2^) + (0.0379*P*)^2^ + 1.6353*P*] where *P* = (*F*_o_^2^ + 2*F*_c_^2^)/3
3785 reflections	(Δ/σ)_max_ = 0.001
172 parameters	Δρ_max_ = 0.46 e Å^−^^3^
0 restraints	Δρ_min_ = −0.53 e Å^−^^3^

### Special details

**Table:**

Geometry. All e.s.d.'s (except the e.s.d. in the dihedral angle between two l.s. planes) are estimated using the full covariance matrix. The cell e.s.d.'s are taken into account individually in the estimation of e.s.d.'s in distances, angles and torsion angles; correlations between e.s.d.'s in cell parameters are only used when they are defined by crystal symmetry. An approximate (isotropic) treatment of cell e.s.d.'s is used for estimating e.s.d.'s involving l.s. planes.
Refinement. Refinement of *F*^2^ against ALL reflections. The weighted *R*-factor *wR* and goodness of fit *S* are based on *F*^2^, conventional *R*-factors *R* are based on *F*, with *F* set to zero for negative *F*^2^. The threshold expression of *F*^2^ > σ(*F*^2^) is used only for calculating *R*-factors(gt) *etc*. and is not relevant to the choice of reflections for refinement. *R*-factors based on *F*^2^ are statistically about twice as large as those based on *F*, and *R*- factors based on ALL data will be even larger.

### Fractional atomic coordinates and isotropic or equivalent isotropic displacement parameters (Å^2^)

**Table:**

	*x*	*y*	*z*	*U*_iso_*/*U*_eq_	
I1	0.423501 (11)	0.337299 (16)	0.478996 (11)	0.05300 (8)	
O1	0.52198 (11)	0.14887 (18)	0.37743 (10)	0.0540 (5)	
O2	0.07995 (12)	−0.1517 (2)	0.71999 (11)	0.0631 (6)	
N1	0.30564 (14)	0.1095 (2)	0.54533 (12)	0.0460 (5)	
H1	0.3092	0.1935	0.5520	0.055*	
C1	0.45213 (16)	−0.0582 (3)	0.38180 (14)	0.0477 (6)	
H1A	0.4938	−0.1126	0.4052	0.057*	
H1B	0.4621	−0.0650	0.3317	0.057*	
C2	0.46504 (15)	0.0831 (2)	0.40367 (13)	0.0401 (5)	
C3	0.40963 (15)	0.1356 (2)	0.45650 (14)	0.0390 (5)	
C4	0.35334 (15)	0.0597 (2)	0.49300 (12)	0.0378 (5)	
C5	0.34440 (16)	−0.0847 (2)	0.47456 (12)	0.0417 (5)	
H5A	0.2873	−0.1125	0.4844	0.050*	
H5B	0.3816	−0.1360	0.5044	0.050*	
C6	0.36464 (17)	−0.1148 (3)	0.39783 (13)	0.0456 (6)	
C7	0.3655 (2)	−0.2658 (3)	0.38817 (17)	0.0698 (9)	
H7A	0.3780	−0.2866	0.3401	0.105*	
H7B	0.3115	−0.3012	0.4003	0.105*	
H7C	0.4076	−0.3039	0.4181	0.105*	
C8	0.29981 (18)	−0.0539 (3)	0.34872 (15)	0.0585 (7)	
H8A	0.3142	−0.0742	0.3009	0.088*	
H8B	0.2990	0.0403	0.3550	0.088*	
H8C	0.2453	−0.0895	0.3592	0.088*	
C9	0.24994 (15)	0.0393 (2)	0.59096 (12)	0.0381 (5)	
C10	0.16832 (16)	0.0851 (2)	0.59962 (13)	0.0424 (5)	
H10	0.1502	0.1594	0.5751	0.051*	
C11	0.11454 (15)	0.0208 (3)	0.64420 (13)	0.0455 (6)	
H11	0.0605	0.0536	0.6508	0.055*	
C12	0.13951 (16)	−0.0923 (3)	0.67950 (12)	0.0437 (6)	
C13	0.22144 (16)	−0.1371 (3)	0.67234 (13)	0.0437 (6)	
H13	0.2393	−0.2119	0.6965	0.052*	
C14	0.27628 (15)	−0.0696 (2)	0.62894 (13)	0.0432 (5)	
H14	0.3317	−0.0978	0.6252	0.052*	
C15	0.1004 (2)	−0.2714 (3)	0.75369 (18)	0.0698 (9)	
H15A	0.0528	−0.3020	0.7801	0.105*	
H15B	0.1469	−0.2573	0.7849	0.105*	
H15C	0.1154	−0.3364	0.7191	0.105*	

### Atomic displacement parameters (Å^2^)

**Table:**

	*U*^11^	*U*^22^	*U*^33^	*U*^12^	*U*^13^	*U*^23^
I1	0.05114 (12)	0.03804 (11)	0.06982 (15)	−0.00787 (7)	0.00678 (9)	−0.00135 (8)
O1	0.0359 (9)	0.0657 (12)	0.0603 (12)	−0.0075 (8)	0.0092 (9)	0.0037 (9)
O2	0.0516 (12)	0.0801 (16)	0.0577 (12)	−0.0013 (10)	0.0190 (9)	0.0170 (10)
N1	0.0504 (12)	0.0374 (11)	0.0503 (12)	−0.0041 (9)	0.0158 (10)	−0.0014 (9)
C1	0.0425 (13)	0.0544 (15)	0.0463 (14)	0.0056 (12)	0.0054 (12)	−0.0065 (12)
C2	0.0310 (11)	0.0487 (14)	0.0406 (13)	−0.0014 (10)	−0.0019 (10)	0.0016 (11)
C3	0.0388 (12)	0.0349 (12)	0.0432 (13)	−0.0038 (9)	−0.0004 (10)	−0.0004 (10)
C4	0.0368 (12)	0.0384 (12)	0.0381 (12)	−0.0022 (10)	−0.0006 (10)	−0.0005 (10)
C5	0.0459 (14)	0.0373 (12)	0.0419 (13)	−0.0069 (10)	0.0024 (11)	0.0000 (10)
C6	0.0489 (14)	0.0450 (14)	0.0428 (14)	−0.0053 (11)	0.0023 (12)	−0.0077 (11)
C7	0.088 (2)	0.0530 (18)	0.0683 (19)	−0.0100 (17)	0.0057 (18)	−0.0191 (15)
C8	0.0502 (15)	0.075 (2)	0.0503 (16)	−0.0110 (15)	−0.0060 (13)	−0.0034 (14)
C9	0.0406 (12)	0.0369 (11)	0.0370 (12)	−0.0015 (10)	0.0040 (10)	−0.0040 (10)
C10	0.0464 (13)	0.0411 (13)	0.0398 (13)	0.0056 (11)	0.0006 (11)	0.0000 (10)
C11	0.0354 (12)	0.0572 (16)	0.0440 (13)	0.0067 (11)	0.0054 (11)	−0.0043 (12)
C12	0.0440 (13)	0.0552 (15)	0.0318 (12)	−0.0019 (11)	0.0072 (11)	−0.0021 (11)
C13	0.0458 (14)	0.0488 (14)	0.0367 (13)	0.0052 (11)	0.0041 (11)	0.0042 (10)
C14	0.0358 (12)	0.0499 (14)	0.0440 (13)	0.0078 (11)	0.0038 (11)	−0.0008 (11)
C15	0.083 (2)	0.067 (2)	0.0587 (18)	−0.0143 (18)	0.0167 (17)	0.0092 (16)

### Geometric parameters (Å, °)

**Table:**

I1—C3	2.095 (3)	C7—H7A	0.9600
O1—C2	1.230 (3)	C7—H7B	0.9600
O2—C12	1.361 (3)	C7—H7C	0.9600
O2—C15	1.407 (4)	C8—H8A	0.9600
N1—C4	1.350 (3)	C8—H8B	0.9600
N1—C9	1.430 (3)	C8—H8C	0.9600
N1—H1	0.8600	C9—C14	1.382 (3)
C1—C2	1.501 (4)	C9—C10	1.389 (3)
C1—C6	1.537 (4)	C10—C11	1.369 (3)
C1—H1A	0.9700	C10—H10	0.9300
C1—H1B	0.9700	C11—C12	1.385 (4)
C2—C3	1.439 (3)	C11—H11	0.9300
C3—C4	1.369 (3)	C12—C13	1.387 (3)
C4—C5	1.508 (3)	C13—C14	1.382 (3)
C5—C6	1.526 (3)	C13—H13	0.9300
C5—H5A	0.9700	C14—H14	0.9300
C5—H5B	0.9700	C15—H15A	0.9600
C6—C8	1.523 (4)	C15—H15B	0.9600
C6—C7	1.537 (4)	C15—H15C	0.9600
			
C12—O2—C15	118.4 (2)	C6—C7—H7C	109.5
C4—N1—C9	127.7 (2)	H7A—C7—H7C	109.5
C4—N1—H1	116.1	H7B—C7—H7C	109.5
C9—N1—H1	116.1	C6—C8—H8A	109.5
C2—C1—C6	115.0 (2)	C6—C8—H8B	109.5
C2—C1—H1A	108.5	H8A—C8—H8B	109.5
C6—C1—H1A	108.5	C6—C8—H8C	109.5
C2—C1—H1B	108.5	H8A—C8—H8C	109.5
C6—C1—H1B	108.5	H8B—C8—H8C	109.5
H1A—C1—H1B	107.5	C14—C9—C10	119.1 (2)
O1—C2—C3	122.4 (2)	C14—C9—N1	121.7 (2)
O1—C2—C1	120.2 (2)	C10—C9—N1	119.1 (2)
C3—C2—C1	117.4 (2)	C11—C10—C9	120.0 (2)
C4—C3—C2	123.3 (2)	C11—C10—H10	120.0
C4—C3—I1	120.70 (18)	C9—C10—H10	120.0
C2—C3—I1	115.92 (17)	C10—C11—C12	120.9 (2)
N1—C4—C3	122.3 (2)	C10—C11—H11	119.6
N1—C4—C5	118.7 (2)	C12—C11—H11	119.6
C3—C4—C5	119.1 (2)	O2—C12—C11	116.0 (2)
C4—C5—C6	113.3 (2)	O2—C12—C13	124.5 (2)
C4—C5—H5A	108.9	C11—C12—C13	119.4 (2)
C6—C5—H5A	108.9	C14—C13—C12	119.5 (2)
C4—C5—H5B	108.9	C14—C13—H13	120.3
C6—C5—H5B	108.9	C12—C13—H13	120.3
H5A—C5—H5B	107.7	C9—C14—C13	120.9 (2)
C8—C6—C5	111.3 (2)	C9—C14—H14	119.5
C8—C6—C1	110.0 (2)	C13—C14—H14	119.5
C5—C6—C1	107.9 (2)	O2—C15—H15A	109.5
C8—C6—C7	109.5 (2)	O2—C15—H15B	109.5
C5—C6—C7	108.3 (2)	H15A—C15—H15B	109.5
C1—C6—C7	109.7 (2)	O2—C15—H15C	109.5
C6—C7—H7A	109.5	H15A—C15—H15C	109.5
C6—C7—H7B	109.5	H15B—C15—H15C	109.5
H7A—C7—H7B	109.5		
			
C6—C1—C2—O1	−160.3 (2)	C2—C1—C6—C8	71.8 (3)
C6—C1—C2—C3	20.7 (3)	C2—C1—C6—C5	−49.8 (3)
O1—C2—C3—C4	−171.1 (2)	C2—C1—C6—C7	−167.7 (2)
C1—C2—C3—C4	7.9 (4)	C4—N1—C9—C14	53.2 (4)
O1—C2—C3—I1	6.0 (3)	C4—N1—C9—C10	−129.6 (3)
C1—C2—C3—I1	−174.98 (17)	C14—C9—C10—C11	−1.4 (4)
C9—N1—C4—C3	−174.5 (2)	N1—C9—C10—C11	−178.7 (2)
C9—N1—C4—C5	5.2 (4)	C9—C10—C11—C12	−2.0 (4)
C2—C3—C4—N1	175.8 (2)	C15—O2—C12—C11	176.3 (3)
I1—C3—C4—N1	−1.1 (3)	C15—O2—C12—C13	−4.2 (4)
C2—C3—C4—C5	−3.9 (4)	C10—C11—C12—O2	−177.1 (2)
I1—C3—C4—C5	179.19 (17)	C10—C11—C12—C13	3.3 (4)
N1—C4—C5—C6	151.7 (2)	O2—C12—C13—C14	179.2 (2)
C3—C4—C5—C6	−28.6 (3)	C11—C12—C13—C14	−1.3 (4)
C4—C5—C6—C8	−67.4 (3)	C10—C9—C14—C13	3.4 (4)
C4—C5—C6—C1	53.4 (3)	N1—C9—C14—C13	−179.4 (2)
C4—C5—C6—C7	172.1 (2)	C12—C13—C14—C9	−2.0 (4)

### Hydrogen-bond geometry (Å, °)

**Table:**

*D*—H···*A*	*D*—H	H···*A*	*D*···*A*	*D*—H···*A*
C14—H14···O1^i^	0.93	2.39	3.313 (3)	174
N1—H1···I1	0.86	2.71	3.227 (2)	120

Symmetry codes: (i) −*x*+1, −*y*, −*z*+1.
